# Assessment of structural disconnections in gliomas: comparison of indirect and direct approaches

**DOI:** 10.1007/s00429-022-02494-x

**Published:** 2022-05-03

**Authors:** Erica Silvestri, Umberto Villani, Manuela Moretto, Maria Colpo, Alessandro Salvalaggio, Mariagiulia Anglani, Marco Castellaro, Silvia Facchini, Elena Monai, Domenico D’Avella, Alessandro Della Puppa, Diego Cecchin, Maurizio Corbetta, Alessandra Bertoldo

**Affiliations:** 1 Department of Information Engineering, University of Padova, 35131 Padua, Italy; 2 Padova Neuroscience Center, University of Padova, 35129 Padua, Italy; 3 Department of Neuroscience, University of Padova, 35128 Padua, Italy; 4 Neuroradiology Unit, University Hospital of Padova, Padua, Italy; 5 Neurosurgery, Department of NEUROFARBA, University Hospital of Careggi, University of Florence, 50139 Florence, Italy; 6 Department of Medicine, Unit of Nuclear Medicine, University of Padova, Padua, Italy; 7 Venetian Institute of Molecular Medicine, 35129 Padua, Italy

**Keywords:** Structural disconnection, Glioma, Diffusion MRI, Tractography

## Abstract

Gliomas are amongst the most common primary brain tumours in adults and are often associated with poor prognosis. Understanding the extent of white matter (WM) which is affected outside the tumoral lesion may be of paramount importance to explain cognitive deficits and the clinical progression of the disease. To this end, we explored both direct (i.e., tractography based) and indirect (i.e., atlas-based) approaches to quantifying WM structural disconnections in a cohort of 44 high- and low-grade glioma patients. While these methodologies have recently gained popularity in the context of stroke and other pathologies, to our knowledge, this is the first time they are applied in patients with brain tumours. More specifically, in this work, we present a quantitative comparison of the disconnection maps provided by the two methodologies by applying well-known metrics of spatial similarity, extension, and correlation. Given the important role the oedematous tissue plays in the physiopathology of tumours, we performed these analyses both by including and excluding it in the definition of the tumoral lesion. This was done to investigate possible differences determined by this choice. We found that direct and indirect approaches offer two distinct pictures of structural disconnections in patients affected by brain gliomas, presenting key differences in several regions of the brain. Following the outcomes of our analysis, we eventually discuss the strengths and pitfalls of these two approaches when applied in this critical field.

**Supplementary Information:**

The online version contains supplementary material available at 10.1007/s00429-022-02494-x.

## Introduction

Gliomas constitute the most common malignant primary brain tumours in adults, and are associated with significant morbidity and mortality (Ostrom et al. 2014). Gliomas frequently develop from glial cells and, according to the World Health Organization (WHO), they are classified into different histological grades that reflect their malignancy or aggressiveness (Louis et al. 2016, 2021). High-grade gliomas (HGG; grades III and IV) are considered malignant tumours and are treated more aggressively than low-grade gliomas (LGG; grades I and II). Standard treatment for gliomas includes neurosurgical resection followed by concomitant chemotherapy and radiation therapy. In this regard, the major goal in surgical planning is to preserve eloquent brain regions and structural connections while removing most of the tumoral tissue (Ghinda et al. 2018; Castellano et al. 2017; Duffau 2019). Due to the highly infiltrative characteristics of these tumours, the impact of gliomas on the structural connectivity of the brain is complex to assess and is currently an open issue in contemporary neuroscience (Nilsson et al. 2018). Since gliomas commonly extend towards gyri and then spread along the course of white matter (WM) tracts, they can be distinguished not only by their anatomic location, but also by which WM tract they have infiltrated (Glenn et al. 2017). A richer depiction of the topological alterations caused by the tumour on the structural connectome may lead to a better understanding of the complex network dysfunctions the pathology causes (Sporns 2011; Castellano et al. 2017) and could identify cortical regions and WM bundles to be carefully navigated during surgery (Duffau 2019). Techniques which identify and quantify the extent WM damage beyond the location of a focal lesion have recently been gaining popularity in the field of stroke and other pathologies, as they tend to explain functional alterations better than damage to specific grey matter (GM) regions (Griffis et al. 2019; Schotten et al. 2020; Di Vita et al. 2019; Salvalaggio et al. 2020). Translating the use of such techniques in the oncological field requires, however, additional cares as the neoplastic nature of tumours poses some difficult challenges to the investigation of the alteration of WM tracts. Indeed, gliomas do not represent a focal and acute lesion but rather a slowly evolving infiltrative process.

When studying the course of WM pathways through imaging, diffusion MRI (dMRI) is the instrument of choice, as it allows to extract the state and directional information of WM fibres through modelling the random motion of water molecules occurring in the different brain regions. Through dMRI, patterns of WM structural disconnections can be derived exploiting the so-called indirect or direct approaches:

- Indirect approaches bypass the need of a subject specific dMRI dataset by projecting the tumoral lesion of a given patient onto a tract-based atlas to detect which WM pathways are most likely to be affected by the pathology (Foulon et al. 2018). Although indirect methods may be more readily accessible, they currently generalize the glioma to be one lesioned region causing homogeneous effects on WM pathways. In this way, the high heterogeneity of the underlying pathological tissues (i.e., oedema, necrosis, tumoral core comprising different cell dimensions, and cellularity) and their specific effects on axonal bundles is completely disregarded.
- Direct approaches involve the quantification of the brain connectome through tractography algorithms applied on dMRI data. Such methodologies are known to have their own limitations and pitfalls (Jeurissen et al. 2019), but, contrary to indirect approaches, have the undisputed advantage of providing subject-specific quantification of WM bundles.

Given the intrinsic difference between direct and indirect approaches, the aim of this study is to propose a quantitative comparison between them. We apply simple but effective image analyses to evaluate benefits and criticalities of both, highlighting points of agreement and divergence in terms of WM disconnection information that can be derived. Moreover, as the oedematous tissue appears to play a crucial role in defining tissutal regions which are subject to inflammation, favourable pathways for tumour spreading, we additionally investigate how patterns of structural disconnections are quantified when the oedematous tissue is included in the definition of pathological lesions.

## Materials and methods

Forty-four patients suffering from de novo brain tumours have been recruited and acquired at the University Hospital of Padova from July 2017 to March 2021. All procedures were in accordance with the ethical standards of the institutional research committee and with the 1964 Helsinki declaration plus later amendments. All participants provided informed, written consent in accordance with the local University Hospital Institutional Review Board.

### MRI acquisition

Data acquisition was performed with a 3 T Siemens Biograph mMR-PET/MR scanner equipped with a 16-channel head-neck coil. The multi-shell dMRI protocol comprised a total of 100 diffusion weighted images (DWIs) (TR/TE 5355/104 ms; voxel size 2 × 2 × 2 mm^3^; FOV 220 × 220 mm^2^; 68 slices; multiband accelerator factor = 2): 10 images at *b* = 0 s/mm^2^, 30 DWIs at *b* value = 710 s/mm^2^ and 60 DWIs at *b* value = 2855 s/mm^2^. This High Angular Resolution Diffusion Imaging (HARDI) protocol is the optimized two shells Neurite Orientation and Dispersion Density Imaging (NODDI) protocol as described in (Zhang et al. 2012). Each diffusion direction was acquired with reverse phase encoding directions, i.e., anterior–posterior and posterior–anterior directions, for distortion correction purposes.

In addition, the acquisition protocol included anatomical imaging, which comprised a 3D T2-weighted (T2w) Fluid Attenuated Inversion Recovery (FLAIR) image (TR/TE 5000/395 ms; TI 1800 ms; voxel size 1 × 1 × 1 mm^3^; FOV 250 × 250 mm^2^; 160 slices), two 3D T1-weighted (T1w) magnetization-prepared rapid acquisition gradient echo (MPRAGE, TR/TE 2400/3.2 ms; TI 1000 ms; voxel size 1 × 1 × 1 mm^3^; FOV 256 × 256 mm^2^; 160 slices) acquired both before and after contrast agent injection and a T2w image (TR/TE 3200/536 ms; voxel size 1 × 1 × 1 mm^3^; FOV 256 × 256 mm^2^; 160 slices).

### Tumour segmentation and structural pre-processing

The anatomical images of each patient were linearly registered to the patient naïve T1w image with the Advanced Normalization Tools (ANTs (Avants et al. 2011), v. 2.0.1). Employing these images, two masks were manually delineated through the ITK-SNAP software (http://www.itksnap.org/) by an expert neuroradiologist with more than 5 years of experience. The first mask, the *T*, included the tumour core (contrast agent enhancing and non-enhancing regions) and the necrosis, where present. The second mask, the *T* + *O*, was created by adding the oedema area to the *T* mask. In addition, each tumour was labelled by the same neuroradiologist as left, right, or bilateral according to the location of its core and to the mainly involved hemisphere. A detailed description of how the segmentation was performed is provided in Supplementary Material Sect. 2.2 and Supplementary Table 1.

Structural pre-processing was applied to the T1w image of each patient and consisted in bias field correction [N4BiasFieldCorrection (Tustison et al. 2010)], skull-stripping [Multi-Atlas Skull Stripping (Doshi et al. 2013)], tissue segmentation [into GM, WM and cortico-spinal fluid, with the unified segmentation tool (Ashburner and Friston 2005) of SPM12 v. 7771], and diffeomorphic non-linear registration (as implemented in ANTs *SyN* algorithm) to the symmetric MNI152 atlas. The last step was performed excluding the *T* + *O* area from the computation of the cost function as suggested in (Andersen et al. 2010).

For each patient, the *T* and *T* + *O* masks were then mapped into the MNI152 exploiting the estimated diffeomorphic non-linear transformations.

Finally, since direct and indirect disconnection mapping methods differ in the tracking of potential disconnections within subcortical areas, for each patient, we used the AAL3 atlas (Rolls et al. 2020) to segment the following regions: thalamus, caudates, putamen, pallidum, and hippocampus. Such areas were discarded not to bias the subsequent methods’ comparison.

### Disconnection maps’ computation

#### Direct disconnection map

*Diffusion MRI pre-processing* The acquired diffusion weighted volumes were visually inspected to identify and remove those images affected by interslice instabilities (Tournier et al. 2011) which were deemed excessively corrupted for subsequent pre-processing techniques to correct. The rest of the pre-processing was executed in its entirety within the *MRtrix3* Software (Tournier et al. 2019) and was featured by an initial denoising step based on random matrix theory (Veraart et al. 2016), and a subsequent call to the tools *TOPUP* (Andersson et al. 2003) and *eddy* (Andersson and Sotiropoulos 2016) from the *FMRIB Software library* (FSL) for B0 inhomogeneity, eddy current, and motion joint correction.

T1w segmentation results (including GM, subcortical parcellation, lesion, and tumour masks) were registered to the naïve B0 volume using ANTs, by applying an affine transformation previously estimated on the patient’s naïve T1w image.

*Diffusion tractography specifications* The reconstruction of patient’s whole brain tractogram was performed in its entirety within the *MRtrix3* software. We first performed multi-shell multi-tissue spherical deconvolution (Jeurissen et al. 2014) to recover the orientation distribution functions for each voxel. Subsequently, we computed the tractogram by exploiting an Anatomically Constrained Tractography (Smith et al. 2012) approach, using as anatomical constraints a five-tissue-type (5TT) segmented tissue image obtained from the patient’s structural T1w image within the MRtrix *5ttgen* routine and including as fifth tissue (i.e., the pathological tissue) the manually segmented lesion mask. Individual fibre tracking was performed with a *second-order Integration over Fiber Orientation Distributions (FOD)* algorithm (Tournier et al. 2010) with streamline termination criteria values set at software defaults (maximum angle *q* = 45°, FOD amplitude cut-off value of 0.1). The number of generated streamlines for each patient initially amounted to 100 M, which were quantitatively reduced to 10 M via the *Spherical-deconvolution Informed Filtering of Tractograms* framework (Smith et al. 2013).

*Disconnection Maps Computation* For each patient, following the quantification of the diffusion tractogram, we computed two patterns of structural disconnections, the first related to the tumour mask (*T* mask) and the second related to the lesion mask (*T* + *O* mask). The computation consisted in the following two steps:

1. We identified the subset of the streamlines in the tractogram that featured a non-null overlap with the tumoral lesion (respectively, with *T* and with *T* + *O* masks).
2. We built a density map of altered tracts. This map was computed in the naïve space of the tractogram and contains for each voxel the count of how many streamlines with non-null overlap with the tumour/lesion are passing through that voxel. We labelled direct structural disconnection (dSD) maps these voxel-wise frequency maps.

We used in-house MATLAB (ver. 2020b, The Mathworks, Natick, MA) scripts to perform the creation of the dSD maps.

As further volumetric analyses of dSD maps required their binarization, the definition of a threshold to define significant disconnection was necessary. We defined such threshold with the following procedure:

1. Similarly to the dSD map quantification, we computed for each subject in the dMRI space a voxel-wise track-density map (Calamante et al. 2010), this time considering the entirety of the tractogram.
2. We brought the individual track-density maps to the MNI152 space via the previously estimated diffeomorphic transformations.
3. In the common MNI152 space, we computed the population average of the individual maps, omitting the lesioned ROI on a patient-by patient basis.
4. To balance the presence of a higher number of patients with left tumours, we symmetrised the frequency template by flipping (left–right) the obtained map, summing the flipped and non-flipped maps, and dividing by 2.
5. Using the same transform as in 2, we projected back the population-averaged track-density map to the T1w space of each subject. We referred this resulting map as AvgDensity.
6. Finally, we considered the structural disconnection value of voxel with coordinates (*x*,*y*,*z*) as significant if the following criterion was met: dSD(*x*,*y*,*z*)/AvgDensity(*x*,*y*,*z*) > 10%.

Additional investigations were performed also using more stringent or permissive thresholds (i.e., 5%, 15%, 20%, and 25%).

Hence, for each patient, we obtained a mask of significantly disconnected voxels.

#### Indirect disconnection maps’ computation

Indirect structural disconnection (iSD) maps were quantified with the BCB Toolkit v. 4.2 (Foulon et al. 2018; Schotten et al. 2020). In choosing which healthy controls tractography atlas to use within the toolbox, we opted for the extended diffusion dataset provided by the toolkit authors, for which the whole brain tractogram of 180 healthy from the Human Connectome Project’ 7 T controls was quantified [full tractography specification in (Schotten et al. 2016)].

In summary, using the BCB Toolkit, for each patient, the lesion masks (both *T* and *T* + *O*) in the MNI152 space were registered to each control naïve space using affine and diffeomorphic deformations and subsequently used as seed for the tractography in TrackVis (http://trackvis.org). Tractographies from the lesions were transformed in visitation maps, binarized and brought to the MNI152. Finally, the percentage overlap map was computed by summing at each point in the MNI152 space the normalized visitation map of each healthy subject. Hence, in the resulting disconnectome map (i.e., iSD), the value in each voxel considers the tracts’ interindividual variability and indicates a probability of disconnection from 0 to 100% for the given lesion.

As no ad-hoc studies are available regarding the recommended use of iSD maps in tumours, we set the probabilistic threshold to 0.5 as in the software defaults. Additional investigations were performed also using more stringent and permissive thresholds (i.e., 0.7 and 0.3, respectively).

### Metrics of comparison

Having obtained a total of four different SD maps (i.e., two for each methodology, different in terms of the employed input mask: *T* and *T* + *O*), we wanted to compare them both intra-methodology (i.e., same approach, *T* versus *T* + *O* maps) and inter-methodology (i.e., same lesion mask, direct versus indirect maps). Before comparing the maps obtained through the two methodologies, the SD maps were masked, using the lesion mask (*T* or *T* + *O*) employed to generate the SD maps. The analyses described hereafter are based on the masked SD maps.

The framework we employed for their comparison had three simple metrics, useful to quantify different similarity features:

- *The difference in volume (ΔVol)* This metric allowed us to quantify the difference in the extension of alteration which is detected by two disconnection maps.
- *The Dice Similarity index (Dice)* The Sørensen-Dice similarity index is a well-known metric of comparison between digital images and is defined by the following formula:
  $$\mathrm{Dice}= \frac{2 \left(A\cap B\right)}{\left|A\right|+\left|B\right|},$$

where *A*, *B* are the two binary matrices for which the similarity needs to be tested. The Dice index quantifies how similar the shape of structural alterations is between two different approaches.

- *The correlation at the intersection (Corr)* We computed the Pearson correlation in a region of interest defined by the intersection of the two matrices in exam. Unlike the Dice index which evaluates only the similarity between shapes, the correlation analysis considers where the hotspots of alteration are in the two SD maps and measures their spatial agreement.

While the ΔVol and Dice metrics were calculated using the binarized disconnection maps, the Corr index was computed using the thresholded SD maps.

The Dice and the ΔVol indexes were computed both considering the entire disconnection maps and subdividing them into their ipsilateral and contralateral components (bilateral tumours were excluded from this last analysis).

To summarise the obtained indexes, we computed the median value and the 25th/75th percentiles across all subjects for each metric of comparison.

### Statistical analysis

To test for statistically significant differences in the ΔVol*,* both considering intra-method and inter-method results at the whole brain or considering only the ipsilateral or contralateral hemisphere, a Wilcoxon rank sum test (significance level α = 0.05) was employed.

To assess whether there was a linear relationship between the comparison metrics and the extension of the input mask, we performed a correlation analysis (Spearman correlation, significance level α = 0.05) between the three indexes (i.e., ΔVol, Dice, and Corr) and the volume of the input mask, separately for *T* and *T* + *O* masks. To test the sensitivity of the comparison metrics to the set of thresholds used for the dSD/iSD maps, we defined their normalized range of variation (nRV) as

$$\mathrm{nRV}\left(k\right)=100\times \frac{\left|\mathrm{max}\left(k\left({t}_{\mathrm{iSD}},{t}_{\mathrm{dSD}}\right)\right)-\mathrm{min}\left(k\left({t}_{\mathrm{iSD}},{t}_{\mathrm{dSD}}\right)\right)\right|}{\mathrm{max}\left(k\left({t}_{\mathrm{iSD}},{t}_{\mathrm{dSD}}\right)\right)},$$

where $k({t}_{iSD},{t}_{dSD}))$ is the median value across the dataset of the comparison metric between two SD maps, given the indirect (${t}_{iSD}$) and direct (${t}_{dSD}$) thresholds. The investigated thresholds values were ${t}_{\mathrm{dSD}}=$[5%, 10%, 15%, 20%, 25%] and ${t}_{\mathrm{iSD}}=$[0.3, 0.5, 0.7].

## Results

Patient's main demographic and clinical information are summarized in Table 1. A detailed description of the single patient characteristics is provided in the Supplementary Table 2. Overall, according to 2021 World Health Organization classification of tumours of the central nervous system (Louis et al. 2021), 33 patients had a glioblastoma, 1 had an astrocytoma, 3 had a glioneuronal and neuronal tumours, 1 had an oligodendroglioma, 1 had a primary diffuse large B-cell lymphoma, 2 had other types of brain tumour (1 intracranial mesenchymal tumour and 1 non otherwise specified tumour), and 3 had an unclassifiable brain tumour, as they deceased shortly before the surgery or did not underwent neurosurgery. The extent of the *T* + *O* mask ranged between 5.7 and 191.6 cm^3^ (mean value 65.1 cm^3^, std 50.4 cm^3^), whereas the extent of the *T* mask between 0.4 and 155.9 cm^3^ (mean value 46.5, std 39.1 cm^3^).

**Table 1:** Patient's main demographic and clinical information for the cohort of subjects included in this study

Age (years)	59.9 ± 14.6
Gender	
Female (*n*)	20
Male (*n*)	24
Tumour histology	
Astrocytoma (*n*)	1
Glioblastoma (*n*)	33
Glioneuronal and neuronal tumours (*n*)	3
Oligodendroglioma (*n*)	1
Primary diffuse large B-cell lymphoma (*n*)	1
Other (*n*)	2
n.a (*n*)	3
Tumour grade	
Low (*n*)	5
High (*n*)	36
n.a. (*n*)	3
IDH-1 mutation status	
Wild type (*n*)	29
Mutated (*n*)	5
n.a. (*n*)	10
Tumour site	
Left (*n*)	22
Right (*n*)	17
Bilateral (*n*)	5

*n.a.* not available

**Fig.1:**
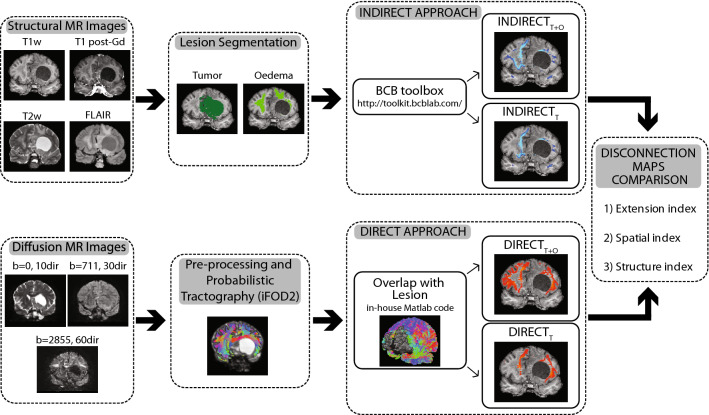
Visualisation of the processing pipeline which led to the computation of the four SD maps (upper row: indirect approach; lower row: direct approach) which were eventually compared

**Fig. 2:**
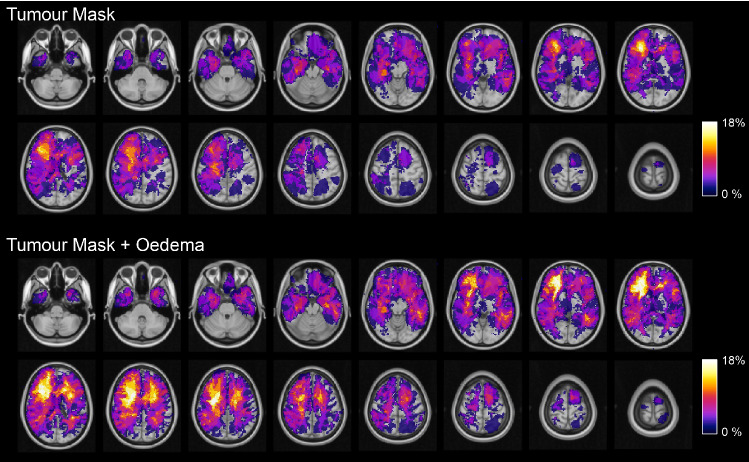
Frequency maps of tumoral lesions in our cohort of patients. In the two upper rows, the lesion was composed by the tumoral core (both enhancing and non-enhancing, and necrotic regions). In the two lower rows, the tumoral lesion mask was composed by the tumour core and the oedematous tissue

Figures 1, 2 shows the frequency maps of the lesions in the patient population. The two reported maps refer to the *T* (first two rows) and to the *T* + *O* masks (second two rows). The distribution is sparse with tumours involving predominantly the right frontal and temporal lobes, with a low spatial overlap (maximum value 17.8% of patients for the *T* mask, and 22.2% of patients for the *T* + *O* mask).

**Fig. 3:**
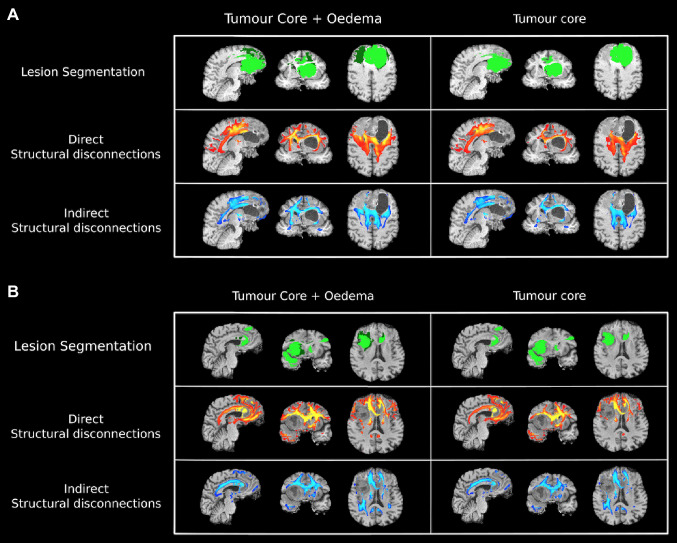
Lesion segmentation (dark green = oedema; green = tumour core), iSD (blue/light-blue), and dSD (red/yellow) maps overlayed on the T1w image for two representative subjects from our cohorts of patients. The SD maps displayed in this figure were obtained after masking for the lesion segmentation mask, reported in the first row of each panel. In the SD maps, the lighter the colour is, the higher the probability/severity of WM disconnection

Figure 3 shows an example of disconnection maps for two representative subjects in our dataset, obtained after masking for the lesion segmentation mask including both the tumour and oedema or just the tumour. In panel A, we can clearly see that the dSD map is able to detect the displacement of axonal fibres in the brain due to the tumoral mass (most apparent in the axial and coronal views). This effect is not detectable by the indirect approach, which inevitably leads to key differences between methodologies (ΔVol(iSD_T+O_,dSD_T+O_) = − 28.05 cm^3^; ΔVol(iSD_T_,dSD_T_) = − 4.8 cm^3^; Dice(iSD_T+O_,dSD_T+O_) = 0.51; Dice(iSD_T_,dSD_T_) = 0.51; Corr(iSD_T+O_,dSD_T+O_) = 0.45; Corr(iSD_T_,dSD_T_) = 0.43)). While the displacement of fibres is less visually appreciable in panel B, differences between approaches are still present (ΔVol(iSD_T+O_,dSD_T+O_) = − 21.1 cm^3^; ΔVol(iSD_T_,dSD_T_) = − 4.1 cm^3^; Dice(iSD_T+O_,dSD_T+O_) = 0.59; Dice(iSD_T_,dSD_T_) = 0.51; Corr(iSD_T+O_,dSD_T+O_) = 0.41; Corr(iSD_T_,dSD_T_) = 0.33)). Looking at the SD maps intra-methodology, i.e., comparing between *T* and *T* + *O* maps, the difference is less self-evident and, apart from a few areas where *T* + *O* maps show more extensive structural disconnections, we find analogous behaviours and morphological features. While ΔVol remains high, suggesting the presence of volumetric differences between the maps, this is well reflected by the Dice and Corr metrics being sensibly higher, both in A. (ΔVol(dSD_T+O_,dSD_T_) = 25 cm^3^; ΔVol(iSD_T+O_,iSD_T_) = 15.17 cm^3^; Dice(dSD_T+O_,dSD_T_) = 0.74; Dice(iSD_T+O_,iSD_T_) = 0.84; Corr(dSD_T+O_,dSD_T_) = 0.79; Corr(iSD_T+O_,iSD_T_) = 0.89)) and in B. (ΔVol(dSD_T+O_,dSD_T_) = 39.36 cm^3^; ΔVol(iSD_T+O_,iSD_T_) = 22.40 cm^3^; Dice(dSD_T+O_,dSD_T_) = 0.75; Dice(iSD_T+O_,iSD_T_) = 0.85; Corr(dSD_T+O_,dSD_T_) = 0.92; Corr(iSD_T+O_,iSD_T_) = 0.86)).

Generalizing Fig. 3 results, Table 2 shows the metrics employed for the comparison in the entire dataset and reports their median and their 25th/75th percentiles. Comparing intra-method results, as expected, we obtained a positive ΔVol which means that the volume of disconnection increased when *T* + *O* is used as input mask (mainly in the whole brain analysis and in the ipsilateral hemisphere). In addition, we obtained a good agreement between the compared maps highlighted both by Dice and by Corr indices, suggesting that in both cases, analogous spatial patterns of disconnection were detected. On the other hand, the inter-method comparison revealed that: (1) the detected volume of disconnection is significantly lower at the whole brain and ipsilateral level for the indirect method when the *T* + *O* mask is used (*p* val, respectively, of 0.005 at whole brain, 0.0008 at ipsilateral level; (2) overall, there is a decrease in the agreement of the disconnection location (whole brain, ipsilateral and contralateral) with a median Dice value of 0.57; (3) there is a poor agreement of the disconnection pattern with a median Corr value of 0.45 and 0.39, respectively, for the *T* and *T* + *O* input masks.

**Table 2:** Median values (with 25th and 75th percentile in squared brackets) for the similarity metrics computed across the patient cohort

	Comparison of intra-method disconnection maps		Comparison of inter-method disconnection maps	
	INDIRECT_T+O_ INDIRECT_T_	DIRECT_T+O_ DIRECT_T_	INDIRECT_T+O_ DIRECT_T+O_	INDIRECT_T_ DIRECT_T_
Extension index				
ΔVol (cm^3^)	9.17 [0.08 29.4]	27.4 [0.11 61.3]	− 25.1 [− 46.2 − 12.3]	− 0.97 [− 21.4 8.1]
ΔVol Ipsilateral (cm^3^)	5.55 [0.07 19.6]	20.2 [0.05 50.3]	− 21.2 [− 33.3 − 10.3]	− 0.43 [− 16 6.3]
ΔVol Contralateral (cm^3^)	2.31 [0 7.63]	2.16 [0 9.83]	− 1.76 [− 11.1 0.62]	− 0.18 [− 4.64 1.58]
Spatial index				
Dice	0.9 [0.62 1]	0.83 [0.39 1]	0.57 [0.52 0.59]	0.46 [0.37 0.57]
Dice ipsilateral	0.9 [0.70 1]	0.85 [0.46 1]	0.57 [0.51 0.6]	0.46 [0.35 0.59]
Dice contralateral	0.86 [0.42 1]	0.88 [0.17 1]	0.53 [0.35 0.61]	0.44 [0 0.59]
Structure index				
Correlation in the intersection	0.91 [0.74 1]	0.87 [0.72 1]	0.45 [0.39 0.50]	0.39 [0.32 0.48]

Each column represents a comparison between two specific SD maps (e.g., the first column is the intra-methodology comparison between the SD maps generated with the *T* and *T* + *O* lesion masks). For further specificity, we additionally divided the analysis of the Dice and ΔVol indexes in the hemispheres ipsilateral and contralateral to the presence of the tumour

As shown in Supplementary Fig. 8, no significant linear relationship was found between the extension of the input map and the similarity of dSD/iSD maps.

**Fig. 4:**
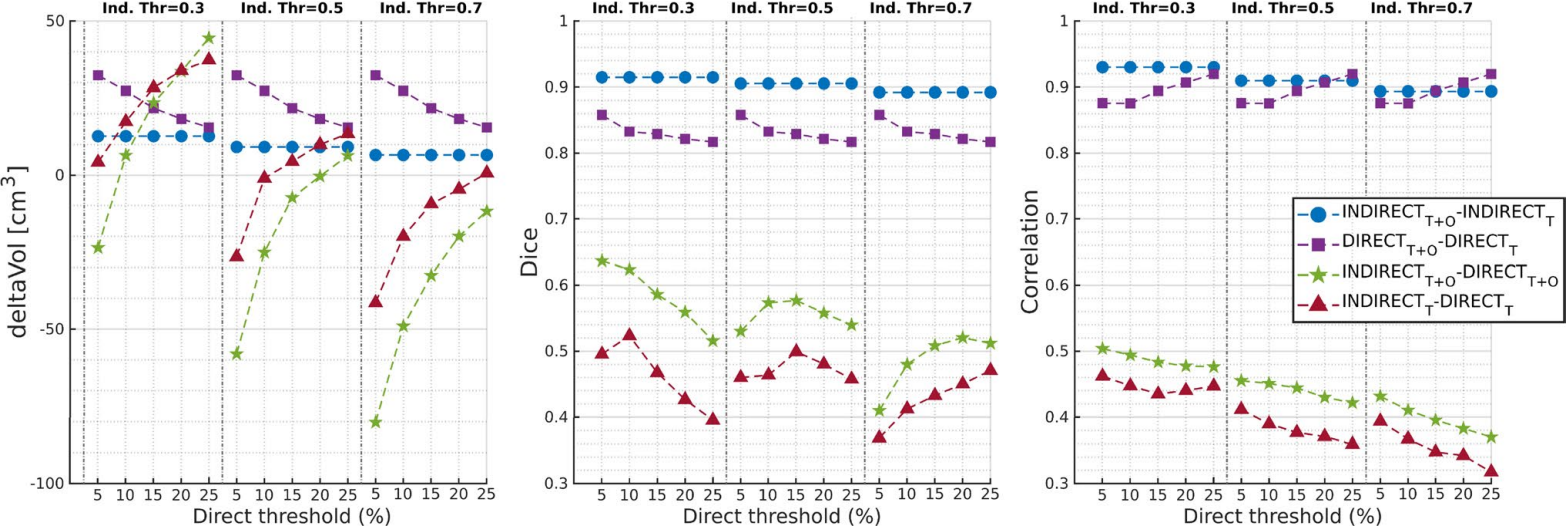
Sensitivity to iSD/dSD thresholds for the similarity metrics in inter- and intra-methodology comparisons. Individual points of the same shape and colour (e.g., green stars, representing the inter-methodology comparison of *T* + *O* maps) are the median values across the dataset for the given index, computed with a different pair of dSD/iSD thresholds

Figure 4 shows the sensitivity of the median of the computed metrics across the dataset to a set of possible thresholds. Considering inter-methodology comparisons (green stars and red triangles in the figure), we found the Vol index to be the most variable across the tested thresholds (nRV_T+O_(ΔVol) = 280.34%, nRV_T_(ΔVol) = 210.34%). Morphology similarities were more consistent (nRV_T+O_(Dice) = 35.58%, nRV_T_(Dice) = 29.53%), as well as the agreement of hotspot locations (nRV_T+O_(Corr) = 26.58%, nRV_T_(Corr) = 31.32%). Shifting to intra-methodology comparisons (blue circles and purple squares in the figure), as expected, we found a more stable situation. Again, volumetric measures were the most variable across thresholds (nRV_T+O_(ΔVol) = 52%, nRV_T_(ΔVol) = 48.24%), and an even higher consistency was found considering the Dice (nRV_T+O_(Dice) = 4.79%, nRV_T_(Dice) = 2.45%), and Corr indexes (nRV_T+O_(Corr) = 4.79%, nRV_T_(Corr) = 3.91%).

## Discussion and conclusions

Throughout the recent years, several warnings have been raised against the use of indirect approaches to investigate the disruption of structural connectivity in neoplastic pathologies, as physio-pathological phenomena such as tissue displacement (Clark et al. 2003) or transneuronal degeneration (Fornito et al. 2015) are not considered by atlas-based methodologies. On the other hand, diffusion tractography techniques may enable the quantification of these effects in individual patients. The framework of analysis we introduced in this article allows to systematically compare patterns of WM disconnections and quantify differences between methodologies. Thus, we here investigated the difference between direct and indirect approaches to quantify patterns of WM disconnections in subjects suffering from brain tumours.

It is important to emphasize that both approaches compute a disconnection map, i.e., a volume that contains the fibres that intersect the tumour core or the tumour core + oedema. However, while the indirect approach defines volumes that contain fibres from a healthy normative atlas going through the regions of interest (tumour, or tumour + oedema) (Foulon et al. 2018), the direct approach defines volumes where the direct measured tractography differs from the pseudo-normative values tractography directly at the patient level.

We compared disconnection maps obtained with either approach using their overall volume, the *Dice* index to evaluate their shape similarities, and finally a correlation analysis aimed to investigate if hotspots of disconnections were identified accordingly. We carried out these analyses both using the tumour core alone and also including the region of surrounding oedema. The latter allows to define the impact of oedematous tissue in the computation of SD maps using either direct or indirect methods.

Interestingly, the volume of disconnection was significantly higher when using the direct vs. the indirect method, especially when the oedema was also considered. This increase occurred particularly in the hemisphere ipsilateral to the tumour, where fibres are most likely to intersect the tumoral lesion. Contralateral fibres, on the other hand, are less likely to intersect the tumoral lesion and the detection of their alterations did not appear to be influenced as much by the oedema. In general, the inclusion of the oedema appeared to drastically alter the extension of ipsilateral patterns of disconnections, but did not severely impact the detection of contralateral alteration.

For what concerns the spatial similarity between the investigated maps, the Dice coefficient revealed good intra-methodology agreement, with both direct/indirect maps featuring high values when testing the difference in terms of inclusion of the oedema in the tumoral lesion. When comparing direct and indirect methodologies however, the Dice coefficient showed lower values indicating a substantial mismatch between the structural disconnection patterns. Such differences remained consistent even when we considered ipsilateral and contralateral hemispheres separately.

Dice values for inter- and intra-methodology maps were well reflected by the Corr index, which revealed whether the localization of hotspots of alterations was concordant between structural disconnection maps. As expected, intra-methodology correlations were strong, with hotspots of alterations having the same spatial location. When we investigated inter-methodology relationships, however, such correspondence was lost, regardless of whether the oedema was included or excluded in the analysis.

Overall, these differences lead us to think that the impact of the inclusion of the oedema in the tumoral lesion has a minor impact than varying between direct/indirect approaches. However, the effect of such choice is still non-negligible and produces sensible changes in the structural disconnection patterns that can be observed.

We initially hypothesized that the size of the tumour would play a pivotal role in the similarity between the patterns of disconnections. However, no significant linear relationship was found between the studied metrics and the tumoral lesion extension (see Supplementary Materials for details of the analysis).

The sensitivity analysis to the thresholds for indirect and direct disconnection maps revealed that volumetric indexes are moderately dependent on such choice, potentially altering the interpretation of its results.

We are aware there are some limitations to our work. First and foremost, the structural disconnection maps must be thresholded to make any inference regarding the volume and shape of the detected disconnections. This choice is non-trivial, and while there are some literature indications (although not specifically in tumour cases) for indirect methodologies (Schotten et al. 2015), thresholding tractograms is an open question in the field of dMRI (Yeh et al. 2021). Volumetric measures of disconnection were significantly impacted by threshold choice, whereas the Dice and Corr indexes were less affected. These observations lead us to raise a warning on the use of volumetric measures when relating structural disconnection features to other quantities of clinical interest. As a second limitation, there are differences in place between the streamline reconstruction of the tract-based atlas of the BCB toolkit, and of the patients belonging to our dataset. While using the same tractography algorithm in both methodologies would indeed eliminate the variability in results due to the different tracking, the purpose of this study was to use the indirect disconnection tools “as is”, and to compare such results with state-of-the-art tracking techniques. Moreover, "voxellizing” the streamlines to the disconnection maps arguably reduces the variability due to diffusion orientation sampling and streamline shapes. Thus, we believe that this discretization step highlights in the final SD maps those differences between the two approaches which are mainly due to dominant physio-pathological effects rather than fibre reconstruction intricacies. As a last note, a few studies have shown that the presence of a tumoral lesion may cause Wallerian degeneration to take place in the surrounding area and at distance from its location (Saksena et al. 2013; Lahrmann et al. 2005; Sawlani et al. 1997). This physio-pathological process represents the inflammatory response of the nervous system to an axonal injury and may significantly impair the permeability of axons in those regions (Pierpaoli et al. 2001), limiting the possibility for tractography algorithms to robustly reconstruct the associated streamlines. Depending on the severity of the degeneration, such WM regions may not be visible to direct approaches, which would consequently fail to detect sites of ongoing pathological alteration.

As a last note, we want to highlight that no tractogram is a perfect representation of brain connectivity, either in pathological or healthy conditions. False-negative (FN)/false-positive (FP) connections are well-known issues in the field, and both indirect and direct methodologies may be affected by them with various degrees (Maier-Hein et al. 2017; Schilling et al. 2019; Sarwar et al. 2019). While probabilistic algorithms (used here in the direct methodology) are better suited to capture peripheral WM pathways, and thus have a reduced number of FN connections, they also may be prone to more incorrect streamlines than deterministic tractography (used here in the indirect methodology) (Maier-Hein et al. 2017). In the attempt of measuring structural disconnections, the choice of which tractogram should be employed still lies with the user, as the FP–FN balance choice should be driven by the specific clinical/research question. We would like to remark, however, that some recent technical advances may play a pivotal role in reducing these issues. As global tractography approaches and filtering procedures like SIFT2 (Smith et al. 2015) and COMMIT (Daducci et al. 2015; Schiavi et al. 2022) see more widespread recognition and usage in the dMRI community, we fully expect streamline-based connectivity inference to gain robustness and biological specificity. Both direct and indirect disconnections methodologies can greatly benefit from these improvements and thus gain relevance in the physio-pathological setting of brain tumours.

In conclusion, with our work, we presented evidence that direct and indirect approaches offer two different pictures of structural disconnections in patients affected by brain tumours. Given these differences, we advise that whenever mass displacement effects appear to be present, direct methodologies should be preferred as they are better suited to account for these morphological and pathological variations. Nevertheless, assessing structural disconnection maps’ predictive value of biological/cognitive progression of glioma patients is vital to addressing the clinical relevance of these techniques. Thus, further studies are needed to answer these unmet demands.

## Supplementary Information

Below is the link to the electronic supplementary material.

Supplementary file1 (DOCX 9682 KB)

## Data Availability

Data used in the present study can be accessed via request to the corresponding author.

## Code availability

The codes and processed data that support the conclusions of this research work can be accessed via request to the corresponding author.
