# Supplementary material for: Assessment of structural disconnections in gliomas: comparison of indirect and direct approaches
Source: Brain Struct Funct. 2022 May 3;227(9):3109–20. doi: 10.1007/s00429-022-02494-x (PMC9653324; doi:10.1007/s00429-022-02494-x)

**Assessment of Structural Disconnections in Gliomas:**

**Comparison of Indirect and Direct Approaches**

Silvestri E.**^†^**, Villani U.**^†^**, Moretto M., Colpo M., Salvalaggio A., Anglani M., Castellaro M., Facchini S., Monai E., D’Avella D., Della Puppa A., Cecchin D., Corbetta M., Bertoldo A.

**^†^** the two authors equally contributed

**SUPPLEMENTARY INFORMATION**

**2. Supplementary Methods:**

***2.1 Segmentation protocol***

Manual segmentation was performed with the ITK-Snap toolbox version 3.8. (www.itksnap.org) slice-by-slice. Structural images (i.e., after contrast agent injection T1w, T2w, and FLAIR) were linearly registered to the individual pre contrast agent injection T1w image. By setting the opacity of the different images, over imposed on each other, segmentation was therefore performed taking into account at the same time the signal intensity across all the images. Moreover, morphological features of the tumour were considered.

Manual segmentation consists in two steps: the identification of the tissues (necrosis, oedema, contrast enhancing and non-contrast enhancing tumour) and the subsequent drawing of the areas. Next, to obtain the tumour mask we merged the necrosis, contrast enhancing and non-contrast enhancing tumour segmented areas. A mistake in identification may have a higher impact on the whole segmentation, that is why our approach was “hierarchical” with three researchers with different level of experience checking each segmentation.

Here an example from the study cohort of step-by-step segmentation:

*STEP 1:* Visualization of pre-contrast T1w, coregistered post-contrast T1w, T2w and FLAIR images.


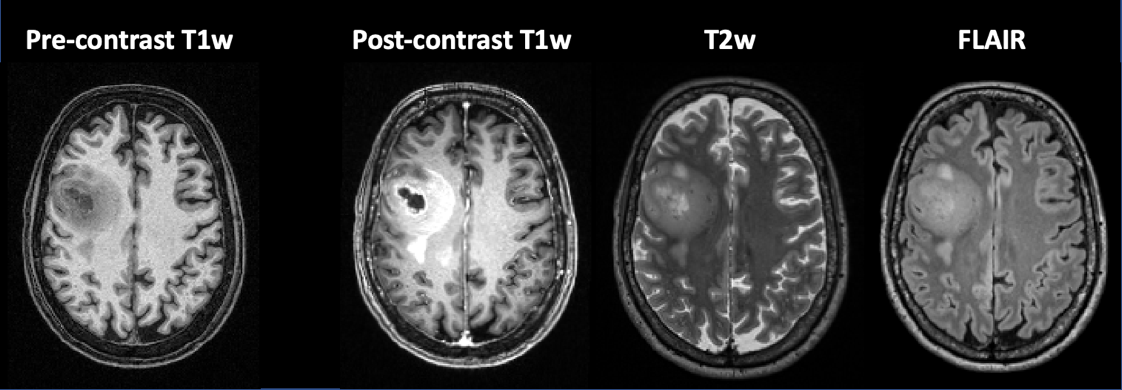

**Supplementary Figure 1**: Axial pre-contrast T1w, post-contrast T1w, T2w and FLAIR sections of a representative GBM.

*
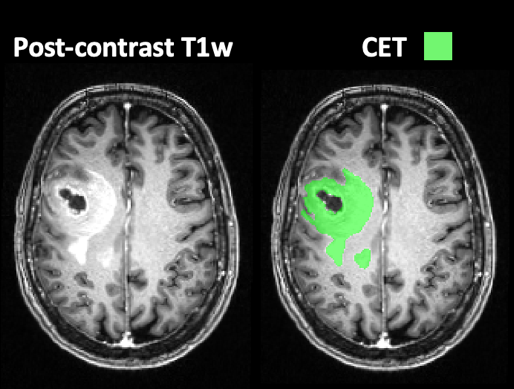
STEP 2:* Contrast-enhancing tumour (CET) segmentation using the post-contrast T1w image.

**Supplementary Figure 2**: Axial post-contrast T1w section before (left) and after (right) CET segmentation (green), in transparency.

*
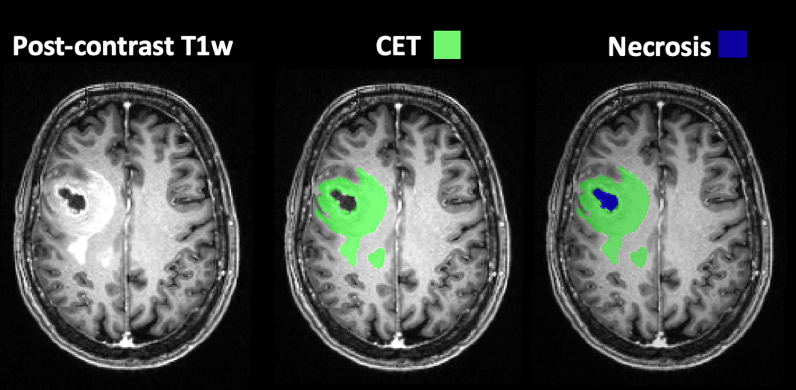
STEP 3:* Necrosis segmentation using the post-contrast T1w image. Typically, necrosis is enclosed by CET.

**Supplementary Figure 3:** Axial post-contrast T1w section, axial post-contrast T1w section with CET segmentation (green), axial post-contrast T1w section with CET and necrosis segmentation label (blue), in transparency.


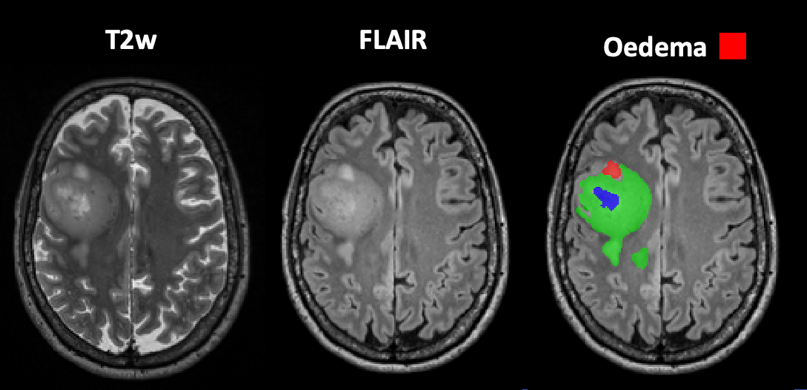
*STEP 4:* Oedema segmentation using the T2w and the FLAIR images.

**Supplementary Figure 4**: Axial T2w and FLAIR sections. On the right, axial FLAIR with CET, necrosis, and oedema (red) segmentations, in transparency.


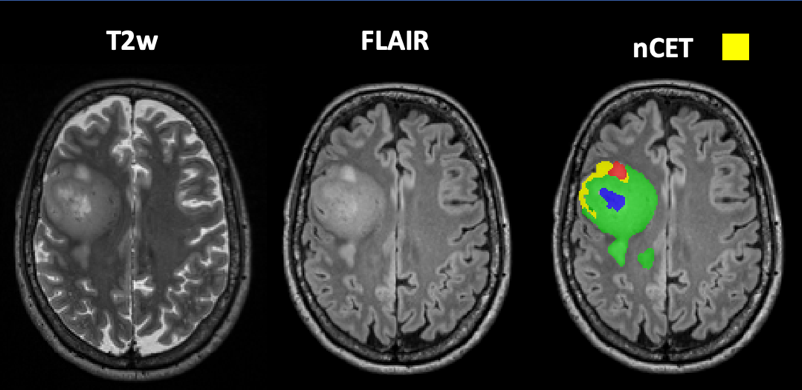
*STEP 5:* Non-contrast-enhancing tumour (nCET) segmentation using the T2w and the FLAIR images.

**Supplementary Figure 5**: Axial T2w and FLAIR sections. On the right, axial FLAIR with CET, necrosis, oedema and nCET (yellow) segmentations, in transparency

All the above-mentioned steps were carried out throughout the whole tumour volume, using all MR sections showing GBM. The two figures below show respectively the different sequences used, with the superimposed complete segmentation and the obtained final segmentation.


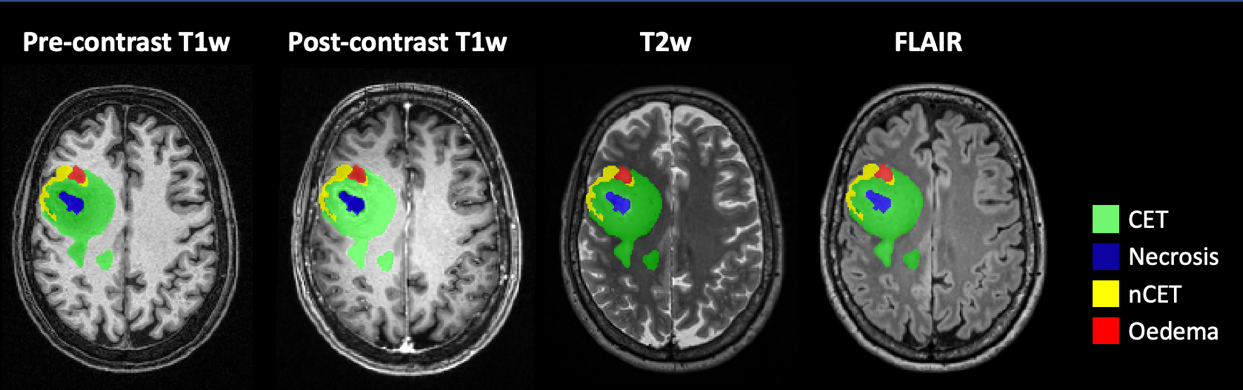


**Supplementary Figure 6**: Axial pre-contrast T1w, post-contrast T1w, T2w and FLAIR sections with all segmentation labels.


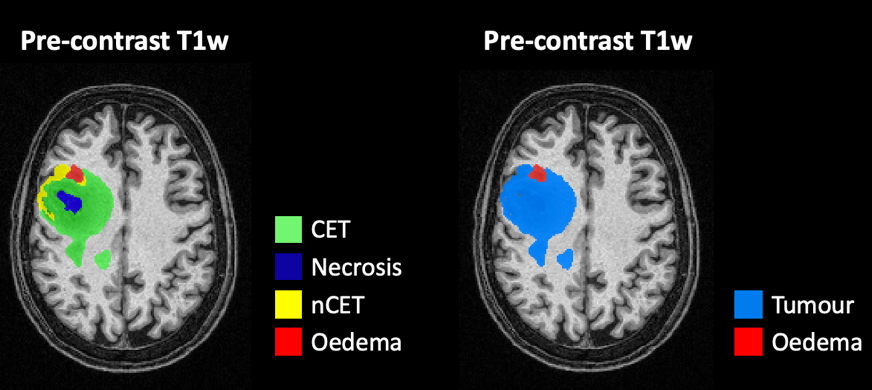


**Supplementary Figure 7**: Axial pre-contrast T1w with all segmentation labels and the final segmentation exploited in this study.

The two most difficult tissues to distinguish are the oedema and non-contrast-enhancing tumour, thus, to discriminate between these areas, the following criteria were used:

|  | Conventional MRI features REPRESENTATIVE of Oedema^1^ | Conventional MRI features representative of non contrast-enhancing tumoUr^1,2^ |
| --- | --- | --- |
| Morphologic criteria | - “Finger-like appearance”, confined to the white matter - Mostly concentric around contrast enhancing tumour | Extension beyond contrast enhancing tumour margin with an eccentric appearance |
| White/grey matter involvement | - Sparing of both cortical and basal nuclei grey matter, possible extension along the internal or external capsule | Involvement of both white and grey matter: typical examples are cerebral cortex, basal nuclei, corpus callosum |
| Mass-effect | - Diffuse or generalized mass-effect | More subtle and localized mass effect, causing architectural distortion. Such anatomical deformations become evident when compared to contralateral intact structures |
| T2-FLAIR intensity | - Marked T2 FLAIR hyperintensity, often fading towards the periphery of edema | Relatively mild T2 FLAIR hyperintensity, compared to edema: this is coherent with the recent finding that T2 FLAIR signal intensity is inversely correlated to cellularity.^3^ |

**Supplementary Table 1:** oedema and non-contrast-enhancing tumour areas discrimination criteria.

***2.2 Relationship between the tumour/lesion size and the similarity of structural disconnection maps***

To assess whether there was a relationship between the comparison metrics and the extension of the input mask, we performed a correlation analysis (Spearman Correlation, significance level 0.05) between *ΔVol,* *Dice* and *Corr* and the volume of the input mask, separately for tumour (T) and tumour plus oedema (T+O) masks. Scatterplots of such analyses were computed to visually inspect such relationships, and the *Simple* *Moving Average* (SMA, 7 points, centerer window) was computed to support the interpretation of possible trends.

**3. Supplementary Results:**

**Supplementary Table 2**: Single patient’s demographical and clinical information. (*Hemi*=involved hemisphere, *T+O volume*=extent of the tumour + oedema segmentation, *T volume*=extent of the tumour segmentation, CC= corpus callosum, F=frontal lobe, L=left, N.A.= not available, O=occipital lobe, P=parietal lobe, R=right, T=temporal lobe, *WT*=wild type, \=not measured)

| **Patient ID** | **Age** | **Gender** | **Histology** | **Classification** | **IDH1** | **Hemi** | **Lobe** | **T+O volume [cm^3^]** | **T volume [cm^3^]** |
| --- | --- | --- | --- | --- | --- | --- | --- | --- | --- |
| 1 | 74 | M | Diffuse glioneuronal tumour | High grade | \ | L | T | 30 | 5 |
| 2 | 25 | F | Oligodendroglioma | Low grade | mutant | L | F | 53.5 | 39.4 |
| 3 | 43 | M | Glioblastoma | High grade | WT | L | F | 81.1 | 0.4 |
| 4 | 56 | F | Intracranial mesenchymal tumour | Low grade | \ | L | F | 6.8 | 6.8 |
| 5 | 69 | M | Glioblastoma | High grade | WT | L | T | 70.1 | 66.2 |
| 6 | 83 | M | N.A. | N.A. | N.A. | L | P-T | 11.7 | 10 |
| 7 | 67 | F | Glioblastoma | High grade | WT | L | P | 19.4 | 6.7 |
| 8 | 36 | M | Glioblastoma | High grade | WT | B | F- CC | 128.9 | 51.4 |
| 9 | 58 | F | Glioblastoma | High grade | WT | R | F-insular + splenium CC + P-O | 76 | 73.9 |
| 10 | 74 | M | Diffuse large B-cell lymphoma | High grade | \ | B | F-P + splenium CC | 27.3 | 8.2 |
| 11 | 83 | F | Glioblastoma epitelioid | High grade | WT | L | F-P | 60.8 | 36.6 |
| 12 | 42 | M | Glioblastoma | High grade | mutant | R | F | 139.2 | 123 |
| 13 | 37 | F | Astrocytoma | Low grade | mutant | L | F | 11 | 11 |
| 14 | 56 | M | Glioblastoma | High grade | mutant | L | F | 127 | 88.8 |
| 15 | 59 | M | Glioblastoma | High grade | WT | R | T + splenium CC | 43.1 | 43.1 |
| 16 | 32 | F | Glioblastoma | High grade | WT | R | Thalamus | 78.3 | 53.3 |
| 17 | 64 | M | Glioblastoma | High grade | WT | B | F-insular R + CC L | 67.9 | 54 |
| 19 | 72 | F | Glioblastoma | High grade | WT | R | P-T | 125.1 | 92.8 |
| 20 | 75 | F | Glioblastoma | High grade | WT | L | T | 80.4 | 8.1 |
| 21 | 68 | M | Glioblastoma | High grade | WT | B | F-T-insular + cingulate cortex + splenium CC | 134.2 | 127.5 |
| 22 | 48 | F | Glioblastoma | High grade | WT | R | T + optic tract | 56.4 | 53.4 |
| 23 | 64 | F | Glioblastoma | High grade | WT | R | F | 12.8 | 9.3 |
| 24 | 46 | F | Glioneuronal neoplasm | High grade | mutant | L | F-insular | 83.1 | 83.1 |
| 27 | 77 | M | Glioblastoma | High grade | WT | L | T | 103 | 85.4 |
| 28 | 57 | M | Glioblastoma | High grade | WT | L | O-T | 50.9 | 43.1 |
| 29 | 49 | F | Glioblastoma | High grade | WT | L | T | 36.8 | 34.5 |
| 30 | 74 | F | Glioblastoma | High grade | WT | L | F | 16.4 | 3.4 |
| 31 | 56 | F | Glioblastoma | High grade | WT | L | T | 6.1 | 6.1 |
| 32 | 49 | M | Glioblastoma | High grade | WT | L | T | 13.1 | 7.8 |
| 34 | 57 | M | Glioblastoma | High grade | WT | R | F | 191.6 | 108.3 |
| 35 | 80 | M | Glioblastoma | High grade | WT | R | O-T | 17.7 | 17.3 |
| 36 | 54 | F | Glioblastoma | High grade | WT | L | T | 25.7 | 24.3 |
| 37 | 36 | F | Not Otherwise Specified | Low grade | \ | R | T | 5.7 | 5.7 |
| 38 | 83 | M | N.A. | N.A. | N.A. | R | P-T-O | 65.3 | 19 |
| 39 | 67 | M | Glioblastoma | High grade | WT | L | F | 49.3 | 48.1 |
| 40 | 51 | M | Multinodular and vacuolating neuronal tumour | Low grade | \ | R | P | 14.9 | 14.9 |
| 41 | 64 | F | Glioblastoma | High grade | WT | R | T-P | 155.9 | 155.9 |
| 43 | 68 | M | Glioblastoma | High grade | N.A. | R | paratrigonal - P | 50.5 | 23.5 |
| 44 | 73 | M | Glioblastoma | High grade | WT | R | F | 122.3 | 44.7 |
| 45 | 57 | F | Glioblastoma | High grade | WT | R | F | 112.4 | 99.5 |
| 46 | 50 | M | Glioblastoma | High grade | N.A. | L | F | 95.1 | 83.3 |
| 47 | 64 | F | Glioblastoma | High grade | WT | R | F | 125.1 | 62.6 |
| 49 | 66 | M | Glioblastoma | High grade | WT | L | F-T- P | 179.2 | 68.5 |
| 50 | 73 | M | N.A. | N.A. | N.A. | B | Splenium CC | 37.3 | 36.9 |

***Relationship between tumour/lesion size and similarity of structural disconnection maps***

Supplementary Figure 1 shows the scatterplot between the extension of the cancerous lesion (both considering only the tumour core (*T*) and including the oedema (*T+O*)) and the similarity metrics. Statistically significant relationships were found for the *Dice* coefficient (ρ_T_ =0.42 p_T_=0.004, ρ_T+O_=0.55 p_T+O_=9.5e-05) and for *ΔVol* (ρ_T_ =0.33 p_T_=0.03). Nevertheless, the simple moving average showed that all three relationships were highly non-linear and heavily influenced by a limited number of small tumours. Indeed, when lesions were larger than 50 cm^3^, the linear trend disappeared due to heavy saturation, leading to no major evidence that the size of the lesion plays a fundamental role in the similarity of direct and indirect structural disconnection maps.

**Supplementary Figure 8**: Scatterplots of tumour/lesion size and the similarity measures. The simple moving average is superimposed in red to highlight the trend of the relationships.


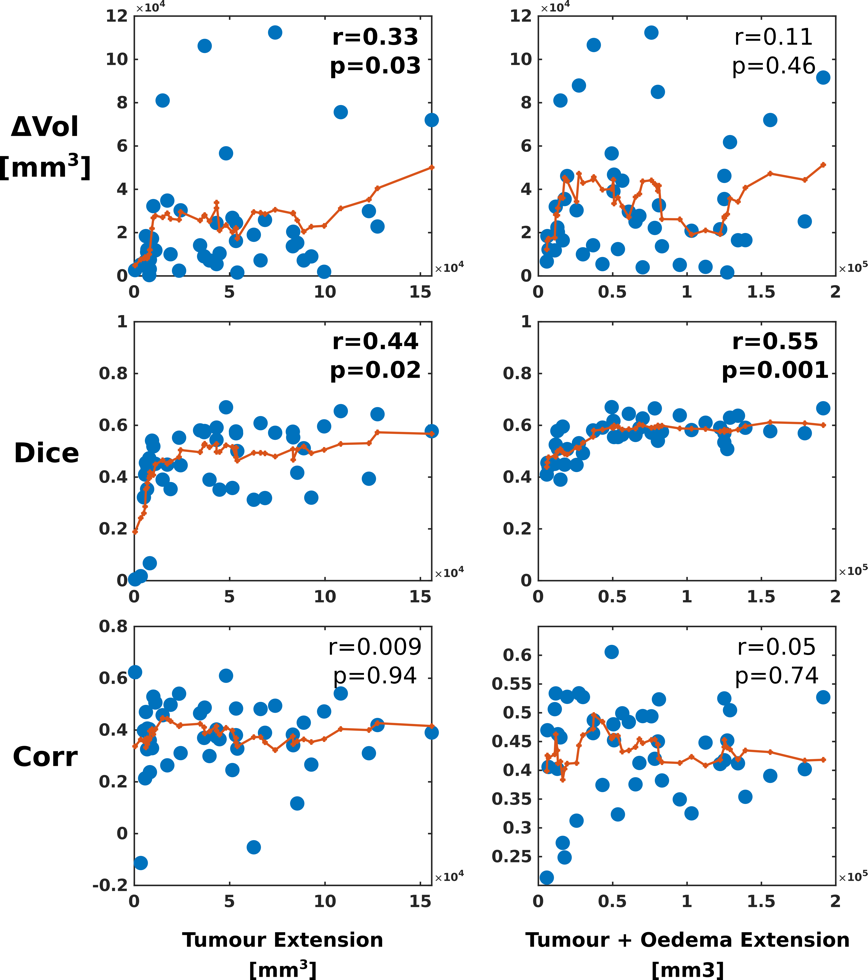

Supplement: Supplementary file 1 — Supplementary file1 (DOCX 9682 KB) [file 429_2022_2494_MOESM1_ESM.docx]
